# Effects of Mild Orthostatic Stimulation on Cerebral Pulsatile Hemodynamics

**DOI:** 10.3389/fphys.2019.00230

**Published:** 2019-03-12

**Authors:** Yuka Ninomiya, Tsubasa Tomoto, Shigehiko Ogoh, Tomoko Imai, Koki Takahashi, Jun Sugawara

**Affiliations:** ^1^Graduate School, Tokyo Ariake University of Medical Health Sciences, Tokyo, Japan; ^2^Human Informatics Research Institute, National Institute of Advanced Industrial Science and Technology, Tsukuba, Japan; ^3^Department of Neurology and Neurotherapeutics, University of Texas Southwestern Medical Center, Dallas, TX, United States; ^4^Institute for Exercise and Environmental Medicine, Texas Health Presbyterian Hospital, Dallas, TX, United States; ^5^Department of Biomedical Engineering, Toyo University, Kawagoe, Japan; ^6^Center for General Education, Aichi Institute of Technology, Toyota, Japan

**Keywords:** orthostatic stress, lower body negative pressure, transcranial Doppler method, pulsatility index, stroke volume

## Abstract

The augmented cerebral hemodynamic pulsatility could lead to the elevated risk of cerebrovascular disease. To determine the impact of an acute orthostatic challenge on a pulsatile component of cerebral hemodynamics, mild lower body negative pressure (LBNP, -30 mmHg) was applied to 29 men. Middle cerebral artery blood flow velocity (MCAv) was measured by transcranial Doppler technique. Stroke volume (SV) was estimated by the Modelflow method with adjustment by the Doppler ultrasound-measured SV at rest. SV, peak and pulsatile MCAv, and pulsatility index were significantly lower during the LBNP stimulation than those at the baseline (e.g., supine resting) (*P* < 0.05 for all), whereas mean arterial pressure and mean MCAv remained unchanged. The change in SV with the LBNP stimulation significantly correlated with corresponding changes in peak and pulsatile MCAv (*r* = 0.617, *P* < 0.001; *r* = 0.413, *P* = 0.026, respectively). These results suggest that pulsatile components of cerebrovascular hemodynamics are dampened during the transient period of orthostatic challenge (as simulated using LBNP) when compared to supine rest, and which is partly due to the modified SV.

## Introduction

There is a gaining recognition that augmented cerebral hemodynamic pulsatility could lead to the elevated risk of cerebrovascular disease. The brain, a high flow organ, is particularly sensitive to excessive pressure and flow pulsatility (Mitchell, 2008). Normally, central elastic arteries (e.g., aorta and carotid artery) dampen blood flow and blood pressure fluctuations by expanding and recoiling vessel wall (called Windkessel function). On the other hand, arterial stiffening is associated with an impaired Windkessel function causes the brain’s chronic exposure to cyclic mechanical forces of cardiac pulsations, promoting abnormalities in the microvascular structure and function (O’Rourke and Safar, 2005; Mitchell et al., 2011).

Furthermore, cardiac ejection *per se* may influence cerebral hemodynamics. In the supine position, stroke volume (SV) is greater than that in the upright position because the reduced hydrostatic pressure facilitates the venous return, and which consequently increases cardiac preload (Rowell, 1986). However, the impact of posture on cerebral hemodynamics, particularly pulsatility, is fully unknown (Bode, 1991). Accordingly, the aim of this study was to determine the impact of an acute orthostatic challenge on a pulsatile component of cerebral hemodynamics. We hypothesized that pulsatile components of cerebrovascular hemodynamics are dampened during the transient period of an orthostatic challenge when compared to supine rest because of the decreased SV.

## Materials and Methods

Twenty-nine healthy men (age = 21 ± 2 year, height = 174 ± 6 cm, body mass = 63.6 ± 8.4 kg, body mass index = 21.0 ± 2.1 kg/m^2^; means ± standard deviation) volunteered for the present study. Each subject provided written informed consent after all of the potential risks and procedures were explained. All experimental procedures and protocol conformed to the Declaration of Helsinki and were approved by the institutional research board of National Institute of Advanced Industrial Science and Technology (No. 2013-434). All subjects were not smokers, had no known cardiovascular or pulmonary disorders, had no history of head injury, and were not taking any prescribed medication known to influence systemic or cerebrovascular function.

The experiment consisted assessment of body composition, cardiac echocardiography test at rest, and measurement of cerebral and systemic hemodynamics at rest and -30 mmHg of lower body negative pressure (LBNP). All measurements were conducted in an environmentally controlled laboratory with a quiet and air-conditioned room (24–25°C). At first, the subjects underwent body composition assessment (e.g., weight and height), and supine rest at least 20 min. Then, echocardiographic examinations were performed to obtain the reference value of SV in the left lateral decubitus position using an ultrasound devise (CX50 × MATRIX, Philips Ultrasound., Bothell, WA, United States) equipped with a multi-frequency probe (2.5-MHz transducer), as we previously reported (Tomoto et al., 2015, 2018). Briefly, Doppler velocity time integral (VTI) was obtained from LV ejection velocity waveform recorded via an apical three-chamber view. SV was computed from multiplying the VTI by the cross-sectional area of the LV outflow tract (LVOT) via a parasternal long axis view, as previously reported (Lewis et al., 1984). LVOT diameter was measured at the base of the aortic valve right after the valve was fully open.

After cardiac data acquirement, the LBNP testing was performed as previously reported (Sugawara et al., 2017; Tomoto et al., 2018). Each participant was placed in a supine position inside the LBNP testing chamber. Once inside, they straddled a wood seat with their feet clear of the base of the chamber and sealed at the level the iliac crest. Following 6 min rest at ambient barometric pressure, negative pressure was gradually induced using a commercially available vacuum and quantified with pressure transducer was invoked. The LBNP test was terminated when the participant completed 4 min at -30 mmHg of lower body pressure. During this experimental protocol, negative pressure was gradually increased and carefully monitored the signs of impending presyncope include dizziness, nausea, profuse sweating, or a rapid change in blood pressure defined as either a decrease in systolic blood pressure by 25 mmHg or a decrease in diastolic blood pressure by 15 mmHg within 1 min. When these signs were reported or observed, the experimental was stopped. From the start of baseline to the end of LBNP stimulation, electrocardiography (ECG) was recorded using a three-lead system for calculation of heart rate (HR) (ML 132 Bio Amp, AD Instruments Inc., Colorado Springs, CO, United States). Radial arterial pressure waveforms were recorded at the left wrist by an applanation tonometry-based blood pressure measurement device (Jentow, Nihon Colin Co., Komaki, Japan), and those were calibrated with oscillometry-derived brachial blood pressure. Middle cerebral artery blood flow velocity (MCAv) was continuously recorded over the right temporal window ipsilaterally using 2-MHz transcranial Doppler probe (EZ Dop; DWL, Sipplingen, Germany). The sampling depth was set from 42 to 55 mm, and the angle of the Doppler probe and the sampling depth were adjusted to optimize the signal quality for each subject according to standard procedures (Tomoto et al., 2015, 2018). Throughout data collection, subjects were instructed to breathe normally. End-tidal CO_2_ (EtCO_2_) was monitored by a metabolic cart equipped with a respiratory gas-analyzing system (AE280S; Minato Medical Science, Tokyo, Japan). Analog signals of ECG, radial arterial pressure, the spectral envelope of MCAv, and EtCO_2_ were continuously stored into a computer using a data acquisition system (PowerLab, AD Instrument Inc., Colorado Springs, CO, United States) throughout the experiment, and analyzed off-line by an analysis software (LabChart, AD Instrument Inc., Colorado Springs, CO, United States). Systolic blood pressures (SBP), diastolic blood pressures (DBP), and pulse pressure (PP) were obtained from the radial arterial pressure waveforms. Time-averaged mean arterial pressure (MAP) was also calculated from the radial arterial pressure waveforms. Systemic hemodynamic pulsatility was assessed as PP/MAP. SV was computed from radial arterial pressure waveforms via the Modelflow method (BeatScope 1.1a, Finapres Medical System BV, Amsterdam, Netherlands) (Wesseling et al., 1993; Sugawara et al., 2003). Modelflow technique could provide reliable information, at least regarding the relative changes in SV during postural alteration (van Lieshout et al., 2003) and submaximal exercise (Wesseling et al., 1993; Sugawara et al., 2003). In addition, the Modelflow technique combined with calibration can quantitatively replace thermodilution estimates. In the present study, in order to minimize estimation error, Modelflow-derived SV was calibrated by Echo-Doppler (LVOT)-derived SV. Cardiac output (CO) was calculated as SV × HR. Systemic vascular conductance (SVC) was obtained as CO/MAP. Time-averaged (mean), peak (systolic), end-diastolic (diastolic), and pulsatile (=peak – end-diastolic) MCAv were calculated from the spectral envelope of MCAv. Pulsatility index (PI) was calculated as pulsatile MCAv/mean MCAv. Cerebrovascular conductance index (CVCi) was calculated as mean MCAv/MAP. Aforementioned systemic and cerebral hemodynamic variables were obtained beat-to-beat and reported averaged values for the last 1 min of each condition (i.e., the baseline and the LBNP condition).

Averaged values for the last 1 min of each condition were calculated and compared. Data were reported as means and standard deviation. Each hemodynamic variable was compared between the baseline and the LBNP condition by a paired *t*-test. Correlation between variables of interests was analyzed by Pearson’s correlation analysis. Statistical significance was set *a priori* at *P* < 0.05.

## Results

No subject showed the signs of impending presyncope during the LNBP testing. Table 1 presents the responses of systemic and cerebral hemodynamics to the LBNP stimulation. SBP, MAP, and mean MCAv remained unchanged during the LBNP stimulation. DBP and HR elevated significantly, and PP, PP/MAP, SV, CO, SVC, CVCi, and EtCO_2_ were significantly reduced during the LBNP stimulation.

**Table 1:** Hemodynamic responses to the lower body negative pressure (LBNP) stimulation.

	Baseline	LBNP	*P*-value
**Cardiovascular hemodynamics**			
HR, bpm	52.9 ± 7.0	59.2 ± 8.6	<0.001
SV, ml	75.0 ± 10.0	63.2 ± 10.5	<0.001
CO, L/min	4.0 ± 0.6	3.8 ± 0.7	0.012
MAP, mmHg	78 ± 7	81 ± 9	0.153
SBP, mmHg	108.9 ± 12.1	108.1 ± 14.9	0.644
DBP, mmHg	61.8 ± 8.1	68.6 ± 11.6	<0.001
PP, mmHg	47.1 ± 8.7	39.5 ± 12.2	0.002
SVC, ml/min/mmHg	52.2 ± 9.4	48.1 ± 10.9	0.007
PP/MAP, ratio	0.61 ± 0.12	0.53 ± 0.18	0.029
**Cerebrovascular hemodynamics**			
Mean MCAv, cm/s	62.6 ± 8.4	59.9 ± 9.5	0.139
CVCi, cm/s/mmHg	0.81 ± 0.41	0.75 ± 0.14	0.016
EtCO_2_, mmHg	39.7 ± 3.4	37.5 ± 3.2	0.003


*Data are mean and standard deviation. HR, heart rate; SV, stroke volume; CO, cardiac output; MAP, mean arterial pressure; SBP, systolic blood pressure; DBP, diastolic blood pressure; PP, pulse pressure; SVC, systemic vascular conductance; MCA, middle cerebral artery blood flow velocity; CVCi, cerebrovascular conductance index; EtCO_2_, end-tidal carbon dioxide*.

**FIGURE 1:**
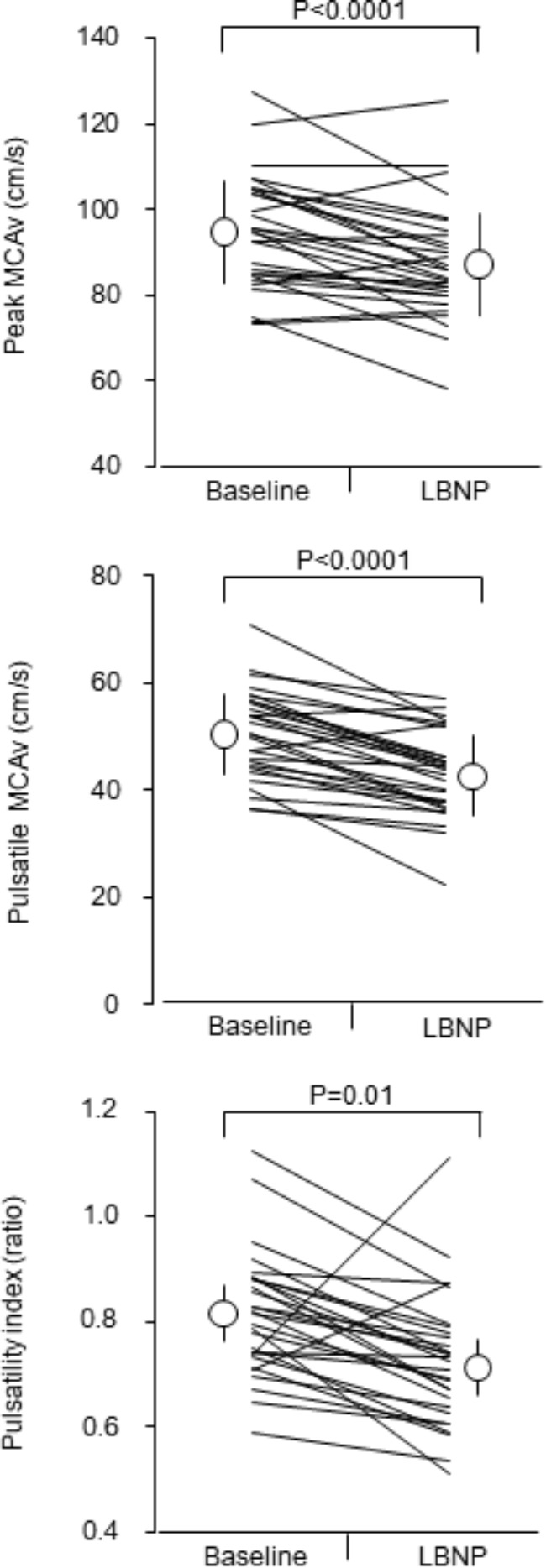
Responses of middle cerebral artery pulsatile hemodynamic measures to mild lower body negative pressure (LBNP) stimulation. Open circles and error bars are mean values and standard deviation. Thin lines indicate individual changes. MCAv = middle cerebral artery blood flow velocity.

Figure 1 depicts responses of middle cerebral artery pulsatile hemodynamic measures to the LBNP stimulation. Peak and pulsatile MCAv (*P* < 0.0001 for both) and PI (*P* = 0.001) were significantly attenuated. The relative change in SV with the LBNP stimulation significantly correlated with corresponding changes in peak and pulsatile MCAv (*r* = 0.617, *P* = 0.0004; *r* = 0.413, *P* = 0.026, respectively) (Supplementary Figure). The relative change in PP with the LBNP stimulation did not significantly correlate with corresponding changes in peak MCAv (*r* = 0.260, *P* = 0.173), pulsatile MCAv (*r* = 0.329, *P* = 0.081), and PI (*r* = 0.117, *P* = 0.546). Likewise, the relative change in PP/MAP with the LBNP stimulation did not significantly correlate with corresponding changes in peak MCAv (*r* = 0.223, *P* = 0.245), pulsatile MCAv (*r* = 0.303, *P* = 0.110), and PI (*r* = 0.271, *P* = 0.156).

## Discussion

The main findings of this study are as follows: first, indices of cerebrovascular pulsatile hemodynamics (e.g., peak and pulsatile MCAv and PI) are significantly higher during the supine than the LBNP condition, whereas mean MCAv, reflecting cerebral perfusion, was maintained. Second, LBNP-induced change in SV correlated with the corresponding changes in peak and pulsatile MCAv. These results suggest that pulsatile components of cerebrovascular hemodynamics are dampened during the transient period of orthostatic challenge (as simulated using LBNP) when compared to supine rest partly due to modified SV. On the other hand, the change in systemic pressure pulsatility (assessed by PP/MAP) was not associated with responses of pulsatile cerebrovascular hemodynamics to the LBNP stimulation.

In this study, we examined whether pulsatile components of cerebrovascular hemodynamics are dampened during the transient period of the orthostatic challenge because of the reduced cardiac ejection (e.g., SV). Addressing this aim, hydrostatic pressure could be a confounding factor which influences cerebral hemodynamics. Therefore, we used LBNP as orthostatic stimulation. In addition, to minimize the change in transmural pressure, we applied mild LBNP stimulation (e.g., -30 mmHg). As expected, SV was significantly larger by 19 % in the supine position presumably due to the enhanced venous return and subsequent increase in cardiac preload (Rowell, 1986), whereas MAP remained unchanged. These systemic hemodynamic responses were similar to a previous study which used the head-up tilt as the orthostatic stimulation (Shaw et al., 2014). However, since the LBNP stimulation may not have the equivalent effects as orthostatic stimulation by the head-up tilt, a further study using the head-up tilt is ideal to clarify this research question.

Importantly, although mild orthostatic stimulation did not influence mean MCAv, the indices of the pulsatile component of cerebrovascular hemodynamics (e.g., peak and pulsatile velocity) were dampened by 8–15 % by the mild LBNP stimulation. The augmented cerebral flow and pressure fluctuations cause several cerebral small vessel diseases (O’Rourke and Safar, 2005; Mitchell et al., 2011; Tarumi et al., 2014). Therefore, our finding may support the notion that the prolonged bedridden develops and accelerates dementia is discussed repeatedly in lay publications. A future study to examine the impact of prolonged bedridden on cerebral hemodynamics and structure is warranted.

Large conduit arteries located cardiothoracic region (e.g., aorta and carotid artery) are highly compliant and dampen intermittent blood flow ejection from the left ventricle as “Windkessel” (a German term meaning elastic reservoir). This role is considered as an important function to protect vulnerable microvasculature, such as the brain, against cyclic mechanical forces of cardiac pulsations (O’Rourke and Safar, 2005). Since we studied young apparent healthy men in this study, it could be thought that the mechanical forces of cardiac pulsations were effectively dampened. Conversely, in individuals who have high arterial stiffness, it is plausible that cyclic mechanical forces of cardiac pulsations are not attenuated appropriately. As the next step, the impact of posture on cerebral hemodynamics, particularly pulsatile component, has to be examined in elderly peoples or hypertensives with the poor Windkessel function of large conduit arteries.

## Conclusion

In conclusion, cerebral pulsatile hemodynamic measures are higher during the baseline (e.g., supine resting) than those during short-term mild orthostatic simulation by LBNP, and that the changes in pulsatile hemodynamic measures relate to LBNP-induced change in SV. These results suggest that pulsatile components of cerebrovascular hemodynamics are dampened during the transient period of orthostatic challenge (as simulated using LBNP) when compared to supine rest partly due to modified SV.

## Author Contributions

JS and SO conceived and designed the research. TT, TI, and JS performed the experiments. YN, TT, TI, and JS analyzed the data. YN, TT, TI, SO, KT, and JS interpreted the results of experiments and approved the final version of the manuscripts. YN prepared the figures and drafted the manuscripts.

## Conflict of Interest Statement

The authors declare that the research was conducted in the absence of any commercial or financial relationships that could be construed as a potential conflict of interest.
