# Supplementary material for: Effects of Mild Orthostatic Stimulation on Cerebral Pulsatile Hemodynamics
Source: Front Physiol. 2019 Mar 12;10:230. doi: 10.3389/fphys.2019.00230 (PMC6423479; doi:10.3389/fphys.2019.00230)
Supplement: Supplementary file 1 [file Presentation_1.PPTX]

## Slide 1
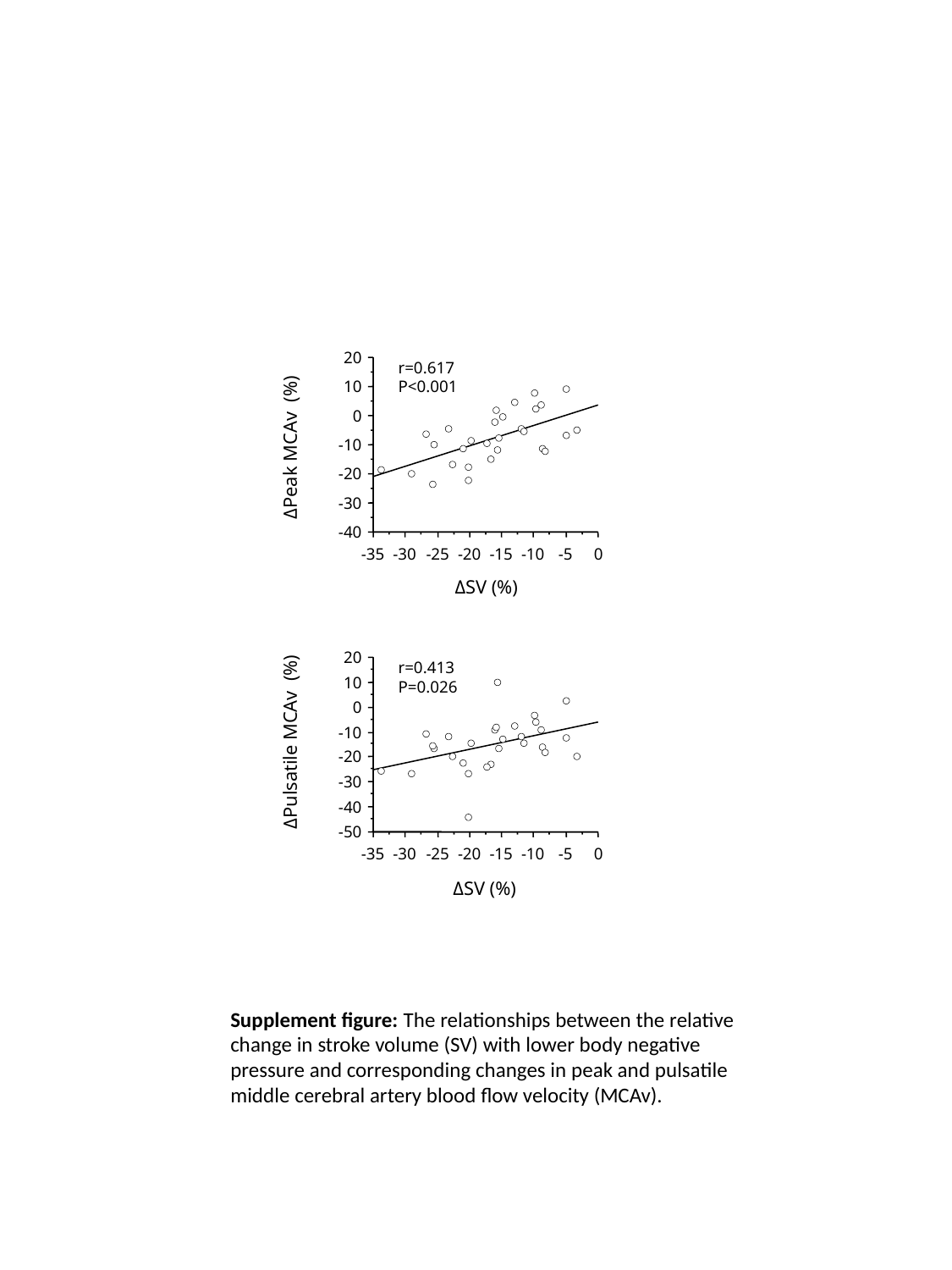

20
r=0.617
P<0.001
10
0
ΔPeak MCAv (%)
-10
-20
-30
-40
-35
-30
-25
-20
-15
-10
-5
0
ΔSV (%)
20
r=0.413
P=0.026
10
0
-10
ΔPulsatile MCAv (%)
-20
-30
-40
-50
-35
-30
-25
-20
-15
-10
-5
0
ΔSV (%)
Supplement figure: The relationships between the relative change in stroke volume (SV) with lower body negative pressure and corresponding changes in peak and pulsatile middle cerebral artery blood flow velocity (MCAv).
